# Molecular determinants for the strictly compartmentalized expression of kainate receptors in CA3 pyramidal cells

**DOI:** 10.1038/ncomms12738

**Published:** 2016-09-27

**Authors:** Sabine Fièvre, Mario Carta, Ingrid Chamma, Virginie Labrousse, Olivier Thoumine, Christophe Mulle

**Affiliations:** 1 Interdisciplinary Institute for Neuroscience, CNRS UMR 5297, University of Bordeaux, 146 rue Léo-Saignat, F-33076 Bordeaux, France

## Abstract

Distinct subtypes of ionotropic glutamate receptors can segregate to specific synaptic inputs in a given neuron. Using functional mapping by focal glutamate uncaging in CA3 pyramidal cells (PCs), we observe that kainate receptors (KARs) are strictly confined to the postsynaptic elements of mossy fibre (mf) synapses and excluded from other glutamatergic inputs and from extrasynaptic compartments. By molecular replacement in organotypic slices from GluK2 knockout mice, we show that the faithful rescue of KAR segregation at mf-CA3 synapses critically depends on the amount of GluK2a cDNA transfected and on a sequence in the GluK2a C-terminal domain responsible for interaction with N-cadherin. Targeted deletion of N-cadherin in CA3 PCs greatly reduces KAR content in thorny excrescences and KAR-EPSCs at mf-CA3 synapses. Hence, multiple mechanisms combine to confine KARs at mf-CA3 synapses, including a stringent control of the amount of GluK2 subunit in CA3 PCs and the recruitment/stabilization of KARs by N-cadherins.

## Abstract (web summary)

Kainate receptors are selectively found at CA3-mossy fibre synapses, although the mechanisms regulating this compartmentalisation have yet to be determined. Here, the authors find KAR segregation is dependent on the amount of GluK2a protein and an interaction between the GluK2 C-terminal domain and N-cadherin.

## Graphical abstract

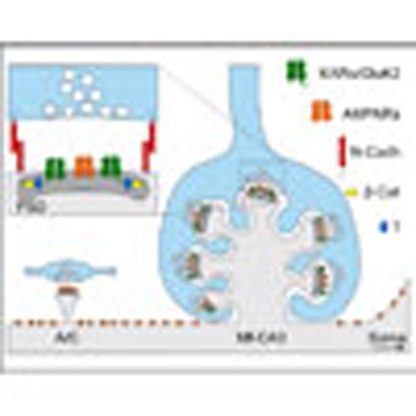

There is an extreme anatomical and functional diversity of synapses connecting different populations of neurons. The segregation of different excitatory connections onto a single neuron is particularly striking in laminated structures such as the hippocampus^1^. CA3 pyramidal cells (PCs) receive three types of glutamatergic inputs, which are precisely positioned along apical and basal dendrites, from the entorhinal cortex (perforant path), from recurrent CA3 collaterals (associational/commissural, A/C fibres) and from the dentate gyrus (DG) through mossy fibres (mf). Glutamatergic synapses from these different inputs greatly vary in their structural and functional properties. Mf inputs make synaptic contacts on proximal dendrites (PDs) of CA3 PCs (in the stratum lucidum) via ‘giant' mf boutons with multiple glutamate release sites facing large postynaptic structures called thorny excrescences (TEs)^2^. In contrast A/C and perforant path synapses are of the single site/single spine type. Mf-CA3 synapses display a wide dynamic range of short-term plasticity and express NMDA (*N*-methyl-D-aspartate) receptor-independent presynaptic forms of long-term depression (LTD) and long-term potentiation (LTP)^3^.

The different families of ionotropic glutamate receptors (iGluRs), AMPA (α-amino-3-hydroxy-5-methyl-4-isoxazole propionic acid) receptors (AMPARs), NMDA receptors and kainate receptors (KARs) exist as multiple subtypes endowed with distinctive properties^4^,^5^. The subcellular segregation of KARs in CA3 PCs provides a striking example of synaptic specification within a single neuronal population. KARs are tetrameric iGluRs composed of combinations of five subunits GluK1–GluK5. KARs regulate the activity of synaptic circuits at presynaptic and postsynaptic sites, through either ionotropic or metabotropic actions^6^,^7^,^8^. KARs are expressed and functional in principal neurons of CA1, DG and CA3, as well as in interneurons^9^. However, postsynaptic KARs activated by synaptic release of glutamate has only been encountered in CA1 interneurons and in CA3 PCs. In CA3 PCs, stimulation of mf inputs but not of A/C inputs evokes excitatory post-synaptic currents (EPSCs) mediated by KARs (KAR-EPSCs), which contain the GluK2 subunit^10^,^11^. In addition, puff application of glutamate above the stratum lucidum, but not the stratum radiatum, activates KAR-mediated inward currents^10^. This segregation of KARs in the stratum lucidum is further supported by kainate-binding studies and by immunocytochemical labelling of GluK2 and GluK5, although these methods do not discriminate between pre- and postsynaptic KARs^12^,^13^. A precise evaluation of the subcellular localization of native KARs in CA3 PCs is made difficult by the lack of high-quality antibodies.

The molecular mechanisms underlying subcellular trafficking of KARs have been explored in heterologous systems and in dissociated neuronal cultures^6^,^7^, albeit in conditions that do not reproduce the strict subcellular segregation observed for native KARs in an *in vivo* situation. The subcellular trafficking and localization of neurotransmitter receptors in neurons are tightly regulated by association with interacting proteins. KAR subunits interact through their cytoplasmic carboxy-terminal domain (CTD) with a large array of proteins involved in the regulation of trafficking to the plasma membrane^14^,^15^,^16^,^17^. In addition, NETO1 and NETO2 (neuropilin tolloid-like 1 and 2) act as auxiliary proteins assembling with KARs to control gating and pharmacology^18^,^19^,^20^,^21^,^22^,^23^. NETO1 contributes to postsynaptic KARs at mf-CA3 synapses^20^,^22^, but the effect of NETO proteins on KAR targeting is unclear^19^,^24^. NETO proteins do not appear to be responsible for the segregation of KARs to mf-CA3 synapses^23^. N-cadherin, which participates in transynaptic complexes to ensure the adhesion between synaptic membranes and organize the underlying multiprotein complex^25^, interacts with GluK2a through its CTD domain^16^. In transfected hereologous cell lines, activation of N-cadherins by ligand-covered latex beads recruits GluK2 to N-cadherin/β-catenin complexes^16^. Whether this interaction plays a role in the trafficking and stabilization of KARs at synaptic sites has not yet been explored.

Here we investigated the mechanisms underlying the confined subcellular expression of KARs in CA3 PCs. Both cell autonomous mechanisms and signals from afferent mfs through transsynaptic-synaptic signalling may be at play. We have used focal glutamate uncaging combined with electrophysiology to precisely characterize the functional expression of KARs and combined this functional approach with a semi-quantitative gene replacement strategy to identify molecular mechanisms for the subcellular segregation of KARs in CA3 PCs.

## Results

### Functional mapping of KARs in CA3 PCs

**Figure 1:**
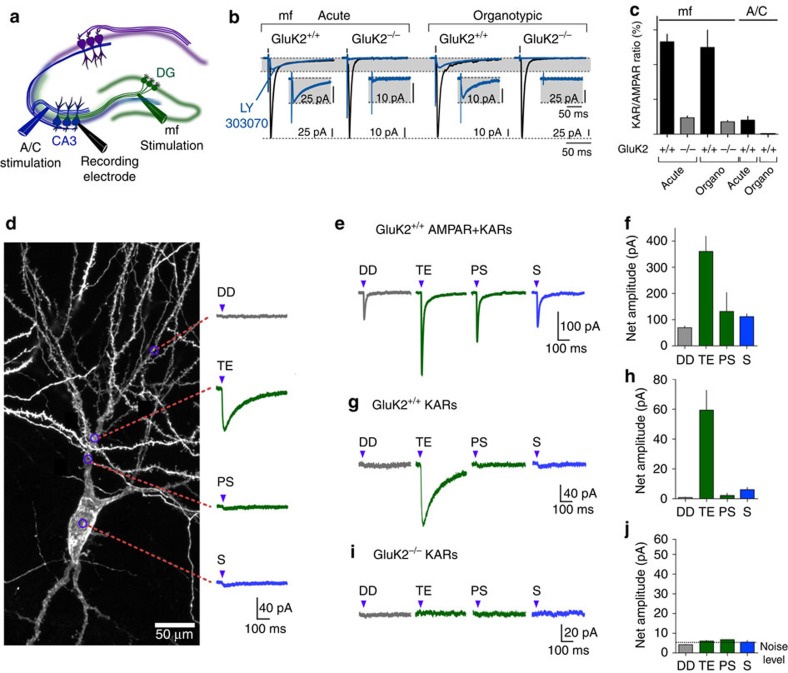
Compartimentalized expression of KARs in CA3 PCs. (**a**) Schematic experimental setting. (**b**) Voltage-clamp recordings performed on CA3 PCs from acute and organotypic slices prepared from GluK2^+/+^ and GluK2^−/−^ mice, in the presence of bicuculline (10 μM), D-AP5 (50 μM) and a low concentration of NBQX (150 nM), to limit polysynaptic activity. In all, average of 30 responses evoked at 3 Hz in the absence and in the presence of 25 μM LY303070, to isolate the mf-CA3 AMPAR-EPSCs and KAR-EPSCs, respectively. Insert, enlargements of isolated KAR-EPSCs. (**c**) Summary graphs for the relative amplitude of KAR-EPSCs versus AMPAR-EPSCs obtained in acute and organotypic slices prepared from GluK2^+/+^ and GluK2^−/−^ mice. Mf KAR/AMPA ratio, acute GluK2^+/+^: *n*=6 cells; organotypic GluK2^+/+^: *n*=8 cells; acute GluK2^−/−^: *n*=5 cells; organotypic GluK2^−/−^: *n*=12 cells; A/C KAR/AMPA ratio: acute GluK2^+/+^: *n*=7 cells; organotypic GluK2^+/+^: *n*=7 cells; 27 mice. (**d**) Confocal image of a CA3 PC transfected with GFP illustrating glutamate uncaging spots (MNI glutamate, 500 μM) and corresponding evoked KAR currents. Purple arrowheads represent time of uncaging (405 nm, 2 ms). Average of five responses evoked at 0.1 Hz at each uncaging site. (**e**,**f**) Sample traces illustrating currents evoked by glutamate uncaging (bicuculline 10 μM, D-AP5 50 μM and NBQX 150 nM) in GluK2^+/+^ organotypic slices in different compartments of CA3 PCs, soma (S), thorny excrescences (TE), proximal shaft (PS) and distal dendrites (DDs). Summary graph of the average of net amplitude of evoked currents. Uncaging sites: S, *n*=3; TE, *n*=7; PS, *n*=3; DD, *n*=10, from 4 cells; 2 mice. (**g**,**h**) Sample traces illustrating KAR currents evoked by glutamate uncaging in GluK2^+/+^ organotypic slices in different compartments of CA3 PCs. Graph summarizing the average net amplitude of evoked KAR currents. Uncaging sites: S, *n*=7; TE, *n*=10; PS, *n*=5; DD, *n*=10, from 10 cells; 6 mice. (**i**,**j**) Sample traces illustrating the lack of KAR currents evoked by glutamate uncaging in GluK2^−/−^ organotypic slices in different compartments of CA3 PCs. Graph summarizing the average net amplitude of the evoked currents. Uncaging sites: S, *n*=8; TE, *n*=18; PS, *n*=1; DD, *n*=8, from 5 cells; 4 mice.

To address the molecular mechanisms underlying the compartmentalized expression of KARs in CA3 PCs, we used organotypic slice cultures as an *in vitro* system easily amenable to genetic manipulation in single identified neurons^26^. We first compared the functional expression of KARs in acute and in organotypic slices. Minimal stimulation of mfs evoked EPSCs in CA3 PCs, which are substantially blocked by LY303070 (25 μM), a selective antagonist of AMPARs. In both acute and cultured slices, the remaining synaptic current represented KAR-EPSCs, which were abolished in slices from GluK2^−/−^ mice (Fig. 1a–c). In both acute and organotypic slices, KAR-EPSCs can be evoked by stimulation of mfs, but not of A/C fibres (Fig. 1b,c). The KAR/AMPAR ratio is comparable between the two conditions (mf KAR/AMPA ratio, acute GluK2^+/+^: 13.2±1.1%; organotypic GluK2^+/+^: 12.4±2.4%; acute GluK2^−/−^: 2.3±0.2%; organotypic GluK2^−/−^: 1.7±0.1%; A/C KAR/AMPA ratio: acute GluK2^+/+^: 2.0±0.5%; organotypic GluK2^+/+^: 0.1±0.0%; Fig. 1c).

KARs are strictly segregated at mf-CA3 PCs synapses and are absent from A/C inputs in organoypic slices, hence validating the model to study the mechanisms underlying synapse-specific segregation of KARs. To get a more precise evaluation of the subcellular segregation of KARs, we mapped functional KARs in single CA3 PCs using focal photolysis of caged MNI-glutamate (500 μM) using 2 ms pulses of a 405 nm laser^27^. Three days before recording, CA3 PCs were transfected by single-cell electroporation with a complementary DNA expressing green fluorescent protein (GFP) to reveal dendritic morphology of CA3 PCs. The output of the laser filled the back of the objective lens by 90%, to generate a minimal spot of ∼1 μm in the objective focal plane. The minimal laser spot formed at the focus can be seen in 100 μM pyranine solution viewed with the charge-coupled device camera^27^. The laser spot was positioned in three dimensions in reference to the distinct compartments of CA3 PCs. We evaluated the precision of the method by moving the laser spot at different distances away from a dendrite in the stratum radiatum, while recording AMPAR-mediated currents evoked by glutamate uncaging (Supplementary Fig. 1a,b). This provided an estimation for the spatial precision of glutamate uncaging of roughly 5–10 μm in diameter, probably corresponding to the range of glutamate diffusion following photolysis.

We then mapped AMPARs and KARs in distinct compartments of CA3 PCs in organotypic slices (Fig. 1d). In CA3 PCs recorded in the voltage-clamp mode, glutamate uncaging evoked AMPAR-mediated currents in the soma, TEs, and proximal shaft and distal dendrites (Fig. 1e,f). Uncaging on distal dendrites included dendritic shaft and spines, whereas the spot for uncaging on the proximal shaft was positioned to avoid TEs. In striking contrast, KAR currents (in the presence of LY303070 and D-AP5) could only be evoked when the uncaging spot was localized above visually identified TEs (average amplitude 59.4±13.2 pA, *n*=10; Fig. 1g,h). No KAR current could be evoked when the laser spot was either positioned on the soma, on the shaft of PDs or on DDs. No KAR current was evoked in GluK2^−/−^ organotypic slices (Fig. 1i,j) or in any compartment of CA1 PCs (Fig. 1d,e), further indicating that all other iGluRs were adequately blocked. In acute slices, functional mapping of KARs revealed a similar restricted pattern of expression (Supplementary Fig. 1f,g). These functional mapping results highlight a clear distinction between iGluR subtypes. AMPARs are readily expressed at synaptic and extrasynaptic sites, that is, on the soma and dendritic shafts. In contrast, KARs appear excluded from the soma and from the proximal and distal dendritic shafts, and are only activated when the laser is focused on TEs. With the current resolution of laser uncaging we cannot exclude the presence of KARs at perisynaptic sites within the TE, given the small spacing (300–400 nm) between the different postsynaptic densities (PSDs)^28^,^29^.

### Semi-quantitative replacement of GluK2 in GluK2^−/−^ mice

**Figure 2:**
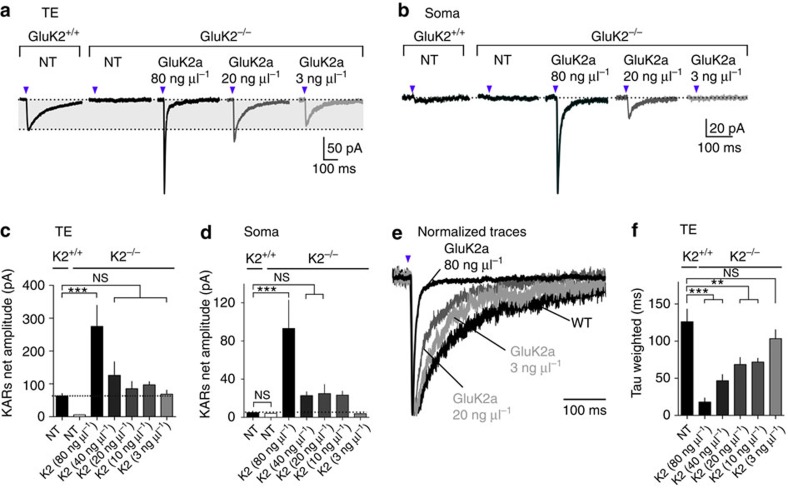
Semi-quantitative replacement of KARs. (**a**,**b**) Sample traces illustrating KAR currents evoked by glutamate uncaging on TEs (**a**) and soma (**b**) of CA3 PCs in organotypic slice cultures prepared from GluK2^+/+^ and GluK2^−/−^ mice not transfected (NT) or transfected with different amounts of GluK2a cDNA (80, 20 and 3 ng μl^−1^). (**c**,**d**) Bar graphs summarizing the average amplitude of KAR currents evoked by glutamate uncaging on TEs (**c**) or on the soma (**d**) in the different conditions (mean amplitude per cell). GluK2^+/+^ NT: *n*=22; GluK2^−/−^ NT: *n*=11; 80 ng μl^−1^: *n*=14; 40 ng μl^−1^: *n*=6; 20 ng μl^−1^: *n*=11; 10 ng μl^−1^: *n*=12; 6 ng μl^−1^: *n*=7; 3 ng μl^−1^: *n*=16; 43 mice. (**e**,**f**) Sample traces of KAR currents evoked by glutamate uncaging shown in **a** and scaled at the peak illlustrating differences in the decay kinetics for various conditions. (**f**) Quantification of the decay rate (tau weighted) of KAR currents evoked by glutamate uncaging for the different conditions. GluK2^+/+^ NT: *n*=7; 80 ng μl^−1^: *n*=7; 40 ng μl^−1^: *n*=6; 20 ng μl^−1^: *n*=10; 10 ng μl^−1^: *n*=12; 6 ng μl^−1^: *n*=8; 3 ng μl^−1^: *n*=6; 43 mice. In all panels, values are presented as mean ±s.e.m. of *n* experiments. Data were compared using one-way analysis of variance followed by Dunnett's multiple comparison test (NS, not significant; *P*>0.05, **P<0.01 and ***P<0.001).

To gain insight into the mechanism of compartmentalization of KARs in CA3 PCs, we used a molecular substitution approach in combination with slice electrophysiology and glutamate uncaging (Fig. 2). In slices prepared from GluK2^−/−^ mice, glutamate uncaging on TEs of CA3 PCs did not evoke any detectable current, as expected. We then rescued functional KARs in GluK2^−/−^ slices using single-cell electroporation of CA3-PCs. A main advantage of single-cell electroporation, with respect to viral infection, is the possibility to control the amount of cDNA transfected, in addition to being able to precisely target CA3 PCs. We transfected various concentrations (3, 10, 20, 40 and 80 ng μl^−1^) of cDNA encoding GluK2a together with cDNA encoding soluble GFP as a reporter (Fig. 2 and Supplementary Fig. 2). Two to 3 days after electroporation of GluK2a cDNA at a concentration as low as 3 ng μl^−1^, KAR currents evoked by focal glutamate uncaging onto TEs could be successfully recorded. The amplitude of KAR currents depended on the amount of cDNA transfected (mean amplitude: 80 ng μl^−1^: 275.2±64.4 pA; 40 ng μl^−1^: 126.1±41.3 pA; 20 ng μl^−1^: 85.3±22.1 pA; 10 ng μl^−1^: 97.3±9.5 pA; 3 ng μl^−1^: 68.7±12.3 pA; Fig. 2a–c). The average amplitude of native KAR currents in wild-type (WT) slices corresponded best to the lowest concentration of GluK2a cDNA (3 ng μl^−1^). Importantly, with higher GluK2a cDNA concentrations, aberrant KAR currents were also evoked with focal glutamate uncaging onto the soma (Fig. 2b–d) and DDs (Supplementary Fig. 2), in stark contrast with the native situation (soma, mean amplitude: 80 ng μl^−1^: 93.2±29.7 pA; 40 ng μl^−1^: 22.8±4.1 pA; 20 ng μl^−1^: 24.9±9.6 pA; 10 ng μl^−1^: 23.3±4.2 pA).

In WT slices, KAR currents displayed slow decay kinetics (mean tau-weighted WT: 126±17 ms), as a probable consequence of the heteromeric composition of native KARs and interaction with auxiliary subunits such as NETOs^18^,^20^,^30^,^31^,^32^. In CA3 PCs of GluK2^−/−^ mice transfected with GluK2a cDNA, the decay kinetics of KAR currents evoked by focal glutamate uncaging onto TEs was much faster than in native conditions for concentrations GluK2a cDNA of 10 ng μl^−1^ and above (mean tau-weighted GluK2a 80 ng μl^−1^: 18.1±5.5 ms; 40 ng μl^−1^: 46.6±8.6 ms; GluK2a 20 ng μl^−1^: 68.6±9.4 ms; GluK2a 10 ng μl^−1^: 71.9±5.1 ms; Fig. 2e,f). The electroporation of 3 ng μl^−1^ GluK2a cDNA restored decay kinetics to values not significantly different from WT (mean tau-weighted GluK2a 3 ng μl^−1^: 103.4±12.0 ms). In addition, KAR currents evoked in the soma for concentrations of GluK2a cDNA of 10 ng μl^−1^ and above also displayed fast decay kinetics (mean tau-weighted GluK2a 80 ng μl^−1^: 16.2±2.1 ms). From a methodological standpoint, these semi-quantitative re-expression experiments clearly show that the amount of cDNA transfected and, consequently, of GluK2a protein is critical for a faithful rescue of KAR subcellular segregation similar to native conditions. The use of a low amount of GluK2a cDNA (3 ng μl^−1^) restored the restricted functional expression of KARs in TEs, whereas transfection of a higher amount of cDNA led to the functional expression of KARs with ectopic localization and aberrant kinetics.

**Figure 3:**
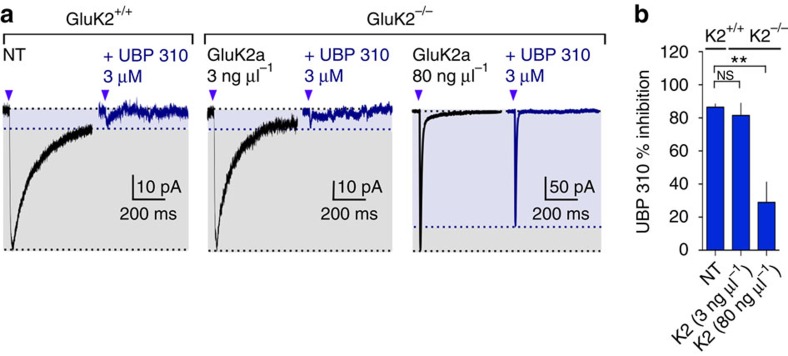
Block of KAR currents by the GluK2/GluK5 antagonist UBP310. (**a**) Sample traces illustrating KAR currents evoked by glutamate uncaging on TEs of CA3 PCs in organotypic slice cultures prepared from GluK2^+/+^ and GluK2^−/−^ not transfected (NT) or transfected with 3 ng μl^−1^ of GluK2a cDNA or 80 ng μl^−1^ in the absence or presence of UBP 310 (3 μM). (**b**) Bar graph summarizing the percentage of inhibition of UBP 310 (3 μM) on the KAR currents evoked by glutamate uncaging. In GluK2^+/+^ NT: *n*=6 cells; 3 ng μl^−1^: *n*=7 cells; 3 ng μl^−1^: *n*=12 cells; 7 mice. Values are presented as mean ±s.e.m. of *n* experiments. Data were compared using one-way analysis of variance followed by Dunnett's multiple comparison test (NS, not significant; *P*>0.05 and **P<0.01).

Recombinant homomeric GluK2 KARs display notably faster deactivation kinetics than heteromeric GluK2/GluK5 when activated by a short (1 ms) pulse of glutamate^31^. Fast decay kinetics of ectopic KAR currents observed in conditions of overexpression of GluK2 may be due to a prevalence of homomeric GluK2. To test this hypothesis, we used UBP310 as an antagonist of recombinant heteromeric GluK2/GluK5 KARs and synaptic KARs, which spares homomeric GluK2 KARs^33^. We observed that in WT slices, KAR currents evoked by glutamate uncaging on TEs was strongly inhibited by 3 μM UBP310 (mean % of inhibition: 86.5±2.0%; Fig. 3) confirming that native KARs in CA3 PCs are likely to be heteromeric KARs. In CA3 PCs from GluK2^−/−^ mice, following transfection of low concentrations of GluK2 cDNA (3 ng μl^−1^), KARs were also largely inhibited by UBP310 (mean % of inhibition: 81.6±7.4%; Fig. 3). In contrast, following transfection of a higher amount of cDNA (80 ng μl^−1^), inhibition of KAR currents evoked by glutamate uncaging on TEs was largely attenuated (mean % of inhibition: 29.0±12.3%) and this inhibition spared the fast decaying component of the response (Fig. 3). These results strongly suggest that following transfection of a high concentration of GluK2a cDNA, ectopic KARs with fast decay kinetics represent a pool of homomeric GluK2 KARs. These experiments also strongly support the notion that native KARs are heteromeric KARs. KAR currents can be successfully rescued in CA3-PCs albeit faithful replacement of KARs in CA3 PCs requires transfection of precise amounts of GluK2a cDNA.

### Synaptic rescue of KARs in GluK2^−/−^ mice

**Figure 4:**
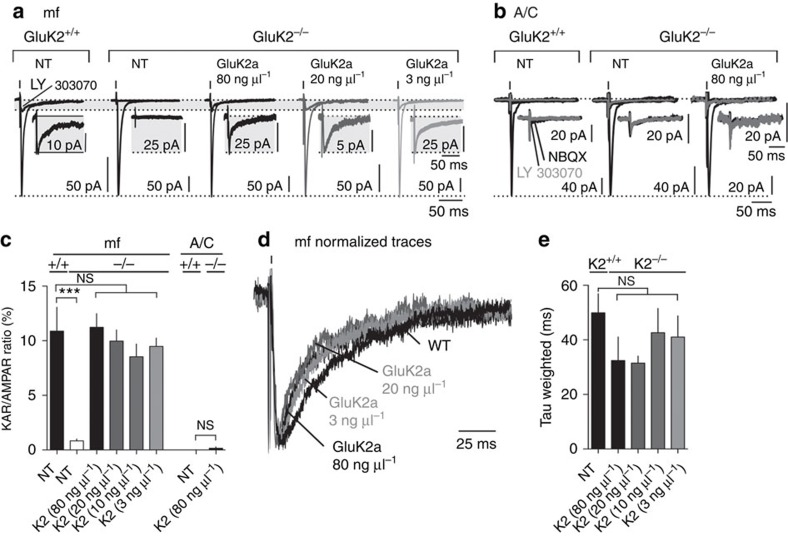
Rescue of synaptic KARs at mf-CA3 synapses. (**a**,**b**) Sample traces illustrating synaptic currents evoked by mf stimulation (**a**) and A/C stimulation (**b**) in organotypic slices prepared from GluK2^+/+^ and GluK2^−/−^ mice, not transfected (NT) or transfected with different amounts of GluK2a cDNA (80, 20 and 3 ng μl^−1^). Experiments were performed in the presence of bicuculline (10 μM), D-AP5 (50 μM) and NBQX (150 nM). Traces represent the average of synaptic currents evoked at 3 Hz in the absence and in the presence of 25 μM LY303070, to isolate the AMPAR and the KAR component, respectively. In the insert are shown enlargements, by the same proportion, of isolated KAR-EPSCs. (**c**) Bar graph summarizing the KAR/AMPAR ratio obtained from the experiments presented in **a** and **b**. Mf stimulation: GluK2^+/+^ NT: *n*=7 cells; GluK2^−/−^ NT: *n*=10 cells; 80 ng μl^−1^: *n*=6 cells; 20 ng μl^−1^: *n*=9 cells; 10 ng μl^−1^: *n*=7 cells; 3 ng μl^−1^: *n*=9 cells. A/C stimulation: GluK2^+/+^ NT: *n*=9 cells; 80 ng μl^−1^: *n*=6 cells; 32 mice. (**d**,**e**) Sample traces of mf-CA3 KAR-EPSCs shown in **a** and scaled at the peak illustrating the lack of difference in the decay kinetics for various conditions. (**e**) Quantification of the decay rate (tau weighted) of KAR-EPSCs from cells transfected with different amount of cDNA encoding for GluK2a. In all panels, values are presented as mean ±s.e.m. of *n* experiments. In **c**, data were compared using one-way analysis of variance followed by Dunnett's multiple comparison test, except for A/C experiments that were compared with an unpaired *t*-test (NS, not significant; *P*>0.05 and ***P<0.001).

We then attempted to selectively rescue synaptic KARs at mf-CA3 synapses in GluK2^−/−^ slices. AMPAR- and KAR-mediated EPSCs were evoked by electrical stimulation of mf and A/C inputs to CA3 PCs (Fig. 4a–c). To normalize the intrinsic variability at mf-CA3 synapses, the relative amplitude of KAR-EPSCs was measured with respect to the amplitude of AMPAR-EPSCs for each cell recorded, as previously described^26^. KAR-EPSCs could be rescued at mf-CA3 synapses with concentrations of GluK2a cDNA of 3 ng μl^−1^ and above. Interestingly, the KAR/AMPAR ratio was comparable to WT for all concentrations of cDNA electroporated (KAR/AMPAR ratio, WT: 10.8±2.1%; GluK2^−/−^ not transfected: 0.8±0.1%; 80 ng μl^−1^: 11.2±1.2%; 20 ng μl^−1^: 9.9±1.0%; 10 ng μl^−1^: 8.5±1.2%; 3 ng μl^−1^: 9.5±0.8%; *P*>0.05; Fig. 4c). Stimulation of A/C inputs did not elicit any KAR-EPSCs neither in WT slices nor in GluK2^−/−^ CA3 PCs transfected with high concentration of GluK2a cDNA (A/C KAR/AMPA ratio, WT: 0.1±0.0%; GluK2a 80 ng μl^−1^: 0.1±0.1%; Student's *t*-test; *P*>0.05). The decay kinetics of KAR-EPSCs were not statistically different between WT and GluK2^−/−^ conditions with various amount of GluK2a cDNA electroporated (mean tau-weighted WT: 49.9±7.0 ms; GluK2 80 ng μl^−1^: 32.4±8.6 ms; 20 ng μl^−1^: 31.5±2.6 ms; 10 ng μl^−1^: 42.6±8.9 ms; 3 ng μl^−1^: 41.0±7.8 ms; *P*>0.05; Fig. 4d,e). Thus, synaptically evoked KARs were reliably rescued at mf-CA3 synapses and were absent from other inputs, mimicking the distribution of native KARs. In addition, whereas the use of high cDNA concentration led to the expression of ectopic extrasynaptic KAR currents of large amplitude, the relative amplitude of KAR-EPSCs did not differ between the different concentrations of cDNA. Although the content of synaptic KARs is precisely controlled independently of the copy number of GluK2a cDNA expressed, the amount of extrasynaptic KARs depends on the concentration of cDNA electroporated. These observations imply that at synaptic contacts an active and specific molecular machinery precisely regulates the amount of KARs, possibly due to a limited number of slots to accommodate synaptic KARs at mf-CA3 synapses.

### Subcellular imaging of recombinant GluK2a in CA3-PCs

**Figure 5:**
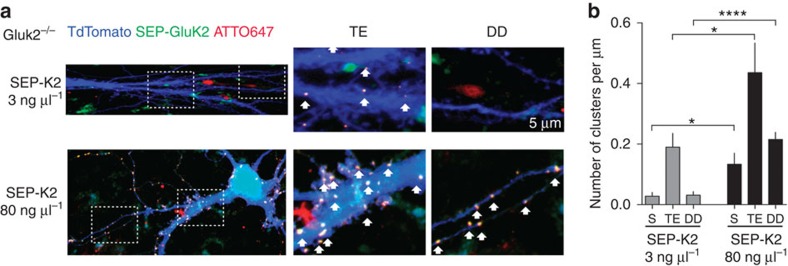
Subcellular distribution of SEP–GluK2a in CA3 PCs. (**a**) Sample images illustrating CA3 neurons electroporated with 3 ng μl^−1^ (top) or 80 ng μl^−1^ (bottom) SEP–GluK2 cDNA (green), TdTomato (blue) and live stained for the SEP moiety using anti-GFP nanobody-ATTO647 (red). Representative confocal *z*-stack projections are shown for each condition. Insets represent characteristic CA3 TEs or DDs. Arrows show the co-localized SEP and anti-GFP nanobody signals that correspond to SEP–GluK2 clusters. (**b**) Average number of clusters per unit length at the soma (S), TE or DD for each condition (3 ng μl^−1^: *n*=11 cells, 3 different experiments, 3 mice; 80 ng μl^−1^: *n*=10 cells, 3 different experiments, 3 mice). In all panels, values are presented as mean ±s.e.m. of *n* experiments. Data were compared using unpaired *t*-test (*P<0.05 and ****P<0.0001).

Visualization of subcellular distribution of KARs neurons is limited by the lack of good antibodies. To circumvent this issue, we expressed GluK2a subunits bearing an amino-terminal SEP tag^34^ in CA3-PCs and labelled them with anti-GFP nanobodies conjugated to photostable organic dyes^35^. These small ligands (∼3 nm) show rapid penetration into thick organotypic tissue in live conditions^36^, allowing robust staining of surface SEP–GluK2a. Specifically, we electroporated SEP–GluK2a at cDNA concentrations of 3 or 80 ng μl^−1^ in CA3 PCs of GluK2^−/−^ slices, thus replacing endogenous KARs by SEP–GluK2a. KAR currents evoked by focal glutamate uncaging after expression of SEP–GluK2a (3 and 80 ng μl^−1^) were of similar amplitudes as that observed when GluK2a-WT was expressed (Supplementary Fig. 3). Moreover, mf-CA3 synaptic KARs were faithfully rescued after electroporation of SEP–GluK2a (3 ng μl^−1^ cDNA; Supplementary Fig. 3). When 3 ng μl^−1^ cDNA concentration was used, SEP–GluK2 clusters detected by confocal microscopy were selectively localized at TEs and were barely detectable in the soma or in DDs (number of clusters per μm length; 3 ng μl^−1^: soma, 0.027±0.012; TE, 0.19±0.045; DD, 0.03±0.036; Fig. 5a,b). In contrast, at high cDNA concentration (80 ng μl^−1^), SEP–GluK2 clusters were abundant in both the soma and DDs, although the highest levels were again detected at TEs (number of clusters per μm length; 80 ng μl^−1^: soma, 0.132±0.036; TE, 0.436±0.097; DD, 0.215±0.023; Fig. 5a,b). Overall, these morphological data corroborate the functional subcellular distribution of KARs and confirm that aberrant ectopic KARs are expressed when a high concentration of cDNA is used.

### GluK2a C terminus stabilizes KARs at mf-CA3 synapses

**Figure 6:**
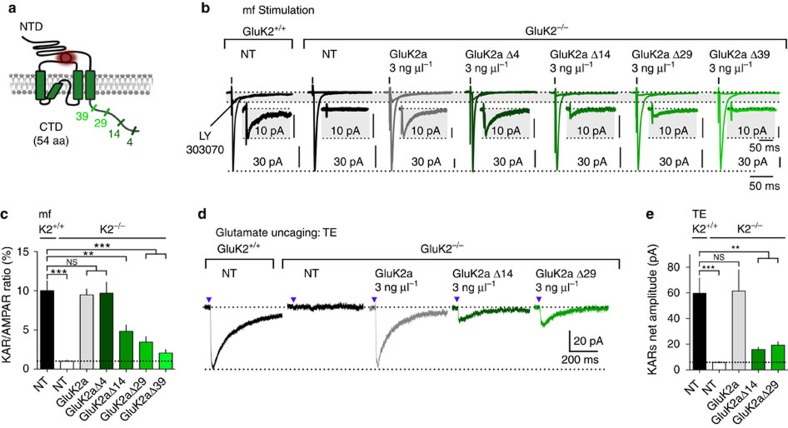
GluK2a C-terminal sequence is necessary for stabilization of synaptic KARs. (**a**) Schematic representation of the GluK2a subunit, illustrating the sites at which the CTD was truncated. (**b**,**c**) Sample traces illustrating synaptic currents evoked by mf stimulation in organotypic slices prepared from GluK2^+/+^ and GluK2^−/−^ mice, not transfected (NT) or transfected with 3 ng μl^−1^ of wt or mutated GluK2a cDNA (Δ4, Δ14, Δ29 and Δ39). Experiments were performed in the presence of bicuculline (10 μM), D-AP5 (50 μM) and NBQX (150 nM). Traces represent the average of synaptic currents evoked at 3 Hz in the absence and in the presence of 25 μM LY303070, to isolate the AMPAR and the KAR component, respectively. In the insert are shown enlargements, by the same proportion, of isolated KAR-EPSCs. (**c**) Bar graphs summarizing KAR/AMPAR ratios obtained from the experiments presented in **b**. GluK2^+/+^ NT: *n*=8 cells; GluK2^−/−^ NT: *n*=8; GluK2^−/−^+GluK2a: *n*=9 cells; GluK2Δ4: *n*=12 cells; GluK2Δ14: *n*=7 cells; GluK2Δ29: *n*=12 cells; GluK2Δ39: *n*=8 cells. 40 mice. (**d**,**e**) Sample traces illustrating KAR-currents evoked by glutamate uncaging on TEs of CA3 PCs in organotypic slice cultures prepared from GluK2^+/+^ and GluK2^−/−^ mice, not transfected (NT) or transfected with 3 ng μl^−1^ of wt or mutated GluK2a cDNA (Δ14 and Δ29). (**e**) Bar graphs summarizing KAR/AMPAR ratios obtained from the experiments presented in **d**. GluK2^+/+^ NT: *n*=11 cells; GluK2^−/−^ NT: *n*=11; GluK2^−/−^+GluK2a: *n*=9 cells; GluK2Δ14: *n*=11 cells; GluK2Δ29: *n*=15 cells; 24 mice. In all panels, values are presented as mean ±s.e.m. of *n* experiments. Data were compared using one-way analysis of variance followed by Dunnett's multiple comparison test (NS, not significant; *P*>0.05, **P<0.01 and ***P<0.001).

The CTD of GluK2 is the target of multiple proteins that modulate the trafficking of KARs to the plasma membrane^6^,^15^,^37^,^38^,^39^. We studied the role of the CTD of GluK2a in the stabilization of KARs at mf-CA3 synapses by reexpressing different truncated forms of GluK2a subunits in CA3 PCs in organotypic slices of GluK2^−/−^ mice (Fig. 6). The cDNAs (3 ng μl^−1^) coding for different GluK2a subunits truncated in the CTD (GluK2aΔ4; GluK2aΔ14; GluK2aΔ29 and GluK2aΔ39) were electroporated in CA3 PCs (Fig. 6a). AMPAR- and KAR-EPSCs were evoked by mf electrical stimulation (Fig. 6b,c). Re-expression of GluK2aΔ4, a GluK2a subunit lacking the PDZ domain-binding motif necessary for the interaction with PSD95, rescued KAR-EPSCs with a relative amplitude comparable to WT (KAR/AMPAR ratio WT: 10.0±1.3%; GluK2aΔ4: 9.7±1.4%), suggesting that this molecular interaction is not critical for the retention of KARs to mf-CA3 synapses. Interestingly, re-expression of both GluK2aΔ14 and GluK2aΔ29 strongly reduced KAR-EPSCs (KAR/AMPAR ratio, GluK2aΔ14: 4.8±0.8%; *P*<0.01; GluK2aΔ29: 3.4±0.7%; *P*<0.001), albeit trafficking of GluK2aΔ14 and GluK2aΔ29 to the plasma membrane is not impaired in dissociated cultured neurons^38^. In addition, the biophysical properties of GluK2a truncated up to the last 29 amino acids do not differ from full-length GluK2a^39^. Hence, the decreased amplitude of KAR-EPSCs may be explained by impaired recruitment or stabilization of mutated KARs at mf-CA3 synapses. Further truncation of the last 39 amino acids (GluK2aΔ39) is known to decrease surface trafficking of GluK2a due to defect in forward trafficking of KARs to the plasma membrane^38^,^39^. Accordingly, expression of GluK2aΔ39 (3 ng μl^−1^) in GluK2^−/−^ mice led to KARs-EPSCs with largely reduced relative amplitude with respect to WT (KAR/AMPAR ratio, WT: 10.0±1.3; GluK2aΔ39: 2.0±0.5; *P*<0.001). KAR currents evoked by glutamate uncaging on TEs following re-expression of GluK2aΔ14 (3 ng μl^−1^) and GluK2aΔ29 displayed largely decreased mean amplitudes as compared with WT (mean amplitude, WT: 59.7±12.0 pA; GluK2aΔ14: 15.9±1.8 pA; GluK2aΔ29: 19.3±2.6 pA; *P*<0.001; Fig. 6d,e). No detectable extrasynaptic KAR currents were however observed in the soma following re-expression of GluK2aΔ14 or GluK2aΔ29 in GluK2^−/−^ mice (Supplementary Fig. 4). Together, these results confirm that the 14 last aminoacids of GluK2a CTD are important for the stabilization of KARs at mf-CA3 synapses.

### N-cadherin is involved in synaptic stabilization KARs

**Figure 7:**
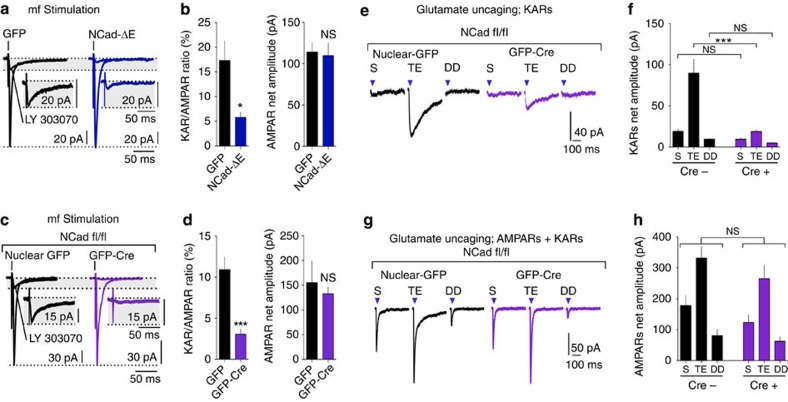
N-cadherin is necessary for the stabilization of synaptic KARs. (**a**,**b**) Averaged synaptic currents evoked by mf stimulation (3Hz) in WT CA3 PCs from organotypic slices transfected with GFP or NCad-ΔE (30 ng μl^−1^) in the absence and in the presence of 25 μM LY303070, to isolate the AMPAR and the KAR component, respectively. Insert enlargements of isolated KAR-EPSCs. Graphs summarizing KAR/AMPAR ratios and net amplitude of AMPAR-EPSCs obtained from experiments presented in **a**. GFP: *n*=6 cells; NCad-ΔE: *n*=6 cells; 7 mice. (**c**,**d**) Averaged synaptic currents evoked by mf stimulation (3Hz) in CA3 PCs from NCad^flox/flox^ organotypic slices transfected with nuclear GFP or GFP-Cre (20 ng μl^−1^), in the absence and in the presence of 25 μM LY303070, to isolate AMPAR and KAR-EPSCs, respectively. Insert, enlargements of isolated KAR-EPSCs. Graphs summarizing KAR/AMPAR ratios and net amplitudes of AMPAR-EPSCs obtained from experiments in **c**. Nuclear-GFP: *n*=7 cells; GFP-Cre: *n*=6 cells; 8 mice. (**e**,**f**) Sample traces illustrating KAR currents evoked by glutamate uncaging (25 μM LY303070) in different compartments (S, TE and DD) of CA3 PCs from NCad^flox/flox^ organotypic slices electroporated either with nuclear-GFP or GFP-Cre. Graph summarizing average net amplitude of evoked KAR currents. Nuclear-GFP; uncaging sites: S, *n*=11; TE, *n*=11; DD, *n*=10. Experiments performed on 11 cells, 6 slices and 4 mice. GFP-Cre; uncaging sites: S, *n*=8; TE, *n*=8; DD, *n*=8. Experiments performed on eight cells, four slices and four mice. (**g**,**h**) Sample traces illustrating AMPAR–KAR currents evoked by glutamate uncaging in different compartments of CA3 PCs (S, TE and DD) from NCad^flox/flox^ organotypic slices electroporated with either nuclear-GFP or GFP-Cre (S, TE and DD). Graph summarizing average net amplitude of evoked AMPAR–KAR currents. Nuclear-GFP; uncaging sites: S, *n*=9; TE, *n*=9; DD, *n*=8. Experiments performed on nine cells, four slices and four mice. GFP-Cre; uncaging sites: S, *n*=8; TE, *n*=8; DD, *n*=8. Experiments performed on eight cells, four slices and four mice. All experiments include bicuculline (10 μM), D-AP5 (50 μM) and NBQX (150 nM). In all panels, values are presented as mean ±s.e.m. of *n* experiments. Data were compared using unpaired *t*-test (NS, not significant; *P*>0.05, *P<0.05 and ***P<0.001).

GluK2a interacts with N-cadherin/β-catenin complexes through its CTD domain; this interaction involves the 14 last amino acids of the CTD and is necessary for N-cadherin-mediated recruitment and aggregation of membrane KARs in heterologous cells^16^. As truncation of this 14 amino-acid sequence strongly decreased KAR-EPSC amplitude, we tested the role of the N-cadherin/β-catenin complex in the stabilization of KARs at mf-CA3 synapses. For this, we first used a dominant-negative approach in organotypic cultures by overexpressing in CA3 cells an N-cadherin protein lacking the entire extracellular domain necessary for homophilic adhesions and transsynaptic adhesion (Ncad-ΔE)^40^. This mutant acts as a dominant-negative construct by sequestering α- and β-catenins^41^, thereby interfering with the binding of GluK2a to endogenous N-cadherin. Under these conditions, we observed a strong reduction in the amplitude of KAR-EPSCs, as quantified by the KAR/AMPAR ratio (mean KAR/AMPAR ratio, GFP: 17.3±3.9%; Ncad-ΔE: 5.8±1.1%; *P*<0.05; Fig. 7a,b). As a control, the net amplitude of AMPAR-EPSCs was not affected by the overexpression of Ncad-ΔE (mean net amplitude, GFP: 114.1±11.5 pA; Ncad-ΔE: 109.8±15.2 pA; Fig. 7b).

Ncad-ΔE may potentially affect signalling through different types of cadherins, for example, cadherin-9, which is also present at mf-CA3 synapses^42^. To show a specific link between KARs and N-cadherin, we deleted endogenous N-cadherin by expression of the Cre recombinase in CA3 PCs from N-cadherin floxed mice (N-Cadherin^fl/fl^)^43^ with a plasmid coding for GFP-cre or for nuclear GFP as a control (Fig. 7c,d). We also observed a marked reduction of KAR-EPSCs in CA3 PCs on knockdown of N-cadherin (10–12 days after electroporation) as quantified by the KAR/AMPAR ratio (mean KAR/AMPAR ratio, nuclear GFP: 10.9±1.6%; GFP-cre: 3.1±0.6%; *P*<0.0001; Fig. 7c,d). The net amplitude of AMPAR-EPSCs at mf-CA3 synapses was not affected by downregulation of N-cadherin (mean net amplitude, nuclear GFP: 155.2±43.7 pA; GFP-cre: 132.5±13.8 pA; Fig. 7d). Similarly, the amplitude and frequency of AMPAR-mediated mEPSCs were not different in CA3 PCs electroporated with either GFP-cre or nuclear GFP (Supplementary Fig. 5). Finally, knockdown of N-cadherin led to a significant decrease in the amplitude of KAR currents mediated by focal glutamate uncaging onto TEs (mean amplitude, nuclear GFP: soma, 19.1±2.9 pA; TE, 89.9±17.1 pA; DD, 9.5±1.0 pA; GFP-cre: soma, 9.4±1.6 pA; TE, 18.9±1.7 pA; DD, 4.7±0.8 pA; Fig. 7e,f). In contrast, the amplitude of AMPAR-mediated currents were not significantly reduced on N-cadherin knockdown with respect to control conditions (mean amplitude, nuclear GFP: soma, 177.5±33.3 pA; TE, 331.2±36.6 pA; DD, 80.2±19.8 pA; GFP-cre: soma, 122.9±24.8 pA; TE, 264.6±42.7 pA; DD, 61.9±15.4 pA; Fig. 7g,h). We obtained similar results when the knockdown of N-cadherin was induced *in vivo* by stereotaxic infection of N-Cadherin^fl/fl^ mice with a virus (AAV 2.9) coding for GFP-Cre, under the control of a synapsin promoter in the CA3 area of the hippocampus. Acute brain slices were prepared and patch-clamp recordings of CA3 PCs were performed 3–4 weeks after infection (Supplementary Fig. 5). Altogether, these results indicate that the N-cadherin/β-catenin complex is involved in the recruitment and/or stabilization of KARs at mf-CA3 synapses.

## Discussion

We show that the strict compartimentalization of KARs at mf-CA3 synapses in CA3 PCs is readily preserved in organotypic slices, providing a much improved model over dissociated cultured neurons, which express functional native KARs, however not synaptically. Similar to that in acute slices, focal glutamate uncaging in CA3 PCs from WT organotypic slices reveals a highly segregated subcellular distribution of functional KARs. KAR currents can only be activated when the uncaging spot is focalized on TEs, but not on the soma, on the proximal dendritic shaft or on distal dendrites and spines. Pathway-specific localization of postsynaptic KARs may be a general feature, which has indeed been reported in pyramidal neurons of the medial entorhinal cortex^44^ and at climbing fibre synapses onto Purkinje cells^45^, also indicating that confined expression of KARs can be observed in the absence of a morphologically specialized postsynaptic element such as TEs. The spatial resolution of our technique, together with the spacing between PSDs of mf-CA3 synapses (*ca*. 500 nm)^28^,^46^, does not allow to exclude the presence of KARs at perisynaptic sites between the various PSDs on TEs. However, functional studies using blockers of glutamate transporters to probe for activation of KARs by glutamate spillover do not favour the presence of KARs at perisynaptic sites^10^,^47^. Nonetheless, our experiments reveal a striking difference between the two related iGluR families AMPARs and KARs. Whereas AMPARs are present at both synaptic and extrasynaptic (soma and dendritic shafts) sites in acute and organotypic slices, KARs are exclusively distributed at a particular synaptic input and absent from extrasynaptic sites. Extrasynaptic AMPARs may serve as a pool of receptors ready to be used for LTP of AMPAR-EPSCs relying on surface diffusion^48^. The lack of a pool of extrasynaptic KARs may explain the inability to induce LTP of KAR-EPSCs^9^, except in artificial conditions where AMPARs are absent^49^.

With this model system in hands, we identified molecular sequences of the major KAR subunit GluK2 required for the strict subcellular segregation observed. We transfected CA3 PCs from GluK2^−/−^ slices to re-express the GluK2a subunit and mutants thereof, based on the fact that GluK2 is an essential component of KARs in CA3 PCs^11^. From a methodological standpoint, we found that transfection of a precise amount of GluK2a cDNA, using single-cell electroporation, was necessary for a faithful restoration of the strict subcellular segregation of KARs. With concentrations of cDNA above 10 ng μl^−1^, re-expression of GluK2a led to the abnormal expression of KARs throughout the somatodendritic compartment of CA3 PCs. Beside the strong methodological warning about the importance of using a semi-quantitative replacement strategy, these results also suggest that the stringent subcellular segregation of native KARs—the exclusion from extrasynaptic sites—depends at least in part on the amount of the GluK2 subunit expressed by CA3 PCs. Hence, in physiological conditions, one can speculate that the amount of GluK2-containing KARs is strongly controlled, either at the transcriptional or postranscriptional level. Not much is known about transcriptional regulation of KARs. At the posttranscriptional level, SUMOylation of GluK2a leads to the removal of KARs from the postsynaptic membrane, leading to a decrease in KAR-mediated synaptic transmission^50^. In addition, the presence of GluK2 in the plasma membrane can be controlled by its interaction with Parkin, which interacts and ubiquitinates GluK2, and as a consequence regulates GluK2 levels and KAR currents in cultured neurons^51^. It would thus be interesting to map KARs in CA3 PCs in the absence of Parkin, or in conditions of reduced SUMOylation of GluK2. The large ectopic KAR currents evoked by brief ultraviolet uncaging flashes observed in CA3 PCs transfected with high concentrations of cDNA displayed fast decay kinetics, which are reminiscent of recombinant homomeric GluK2 KARs^31^ and are very different from native KARs recorded on TEs. The block of ectopic KAR currents by UBP310, an antagonist of heteromeric GluK2/GluK5 receptors but not of homomeric GluK2 (ref. 33), indicates that ectopic KARs are probably homo-tetramers. This suggests that the availability of GluK4 or GluK5 to form heteromers are in controlled and limited amounts in CA3 PCs. Hence, non-controlled overexpression of GluK2 leads to the loss of the strict compartimentalization of KARs in CA3 PCs giving rise to abnormal homomeric GluK2 at extrasynaptic sites.

Re-expression of GluK2a in CA3 PCs of GluK2^−/−^ mice restores expression of KARs at mf-CA3 synapses. Interestingly, the amplitude and kinetics of KAR-EPSCs appear independent of the amount of cDNA transfected, a finding compatible with a synaptic slot hypothesis^48^. Whatever the cDNA concentration, KAR-EPSCs cannot be recorded at A/C synapses. We thus propose that only a limited amount of slots selective for KARs are available at mf-CA3 synapses. Once these slots are occupied, overexpressed KARs invade extrasynaptic sites. The limited slots for KARs correlates with the observation that in physiological conditions, KAR-EPSCs can undergo LTD^26^,^52^,^53^, but not LTP. LTD may correspond to the diffusion of heteromers of GluK2 and/or GluK4 and GluK5 out of synapses, followed by rapid internalization. GluK4 and GluK5 are certainly essential elements in controlling the synaptic amount of KARs^26^,^52^ and removing both GluK4 and GluK5 abolishes KAR-EPSCs^32^.

What constitutes a KAR synaptic slot remains an open question. Possibilities include PDZ-domain-containing proteins such as PSD95, PICK or GRIP^14^,^15^,^37^,^54^, although re-expression of a mutated GluK2a subunit lacking the four last amino acids responsible for the interaction with PDZ domain proteins did not affect KAR-EPSCs. In contrast, removal of the last 14 amino acids of GluK2a strongly had an impact on the amplitude of KAR-EPSCs. Interestingly, these last 14 amino acids are essential for the interaction of GluK2-containing KARs with N-cadherin/β-catenin complexes, albeit this interaction is likely to be indirect^16^ and other proteins could potentially interact with GluK2 through these amino acids. N-cadherins are synaptic adhesion proteins implicated in synapse formation and maturation^55^. Activation of N-cadherins by ligand-covered latex beads recruits GluK2a to N-cadherin/β-catenin complexes in heterologous cells in a manner, which strictly depends on the last 14 aa of GluK2a^15^. Here we demonstrate that overexpression of a dominant-negative N-cadherin or knocking down N-cadherin in slice cultures or *in vivo* strongly reduces KAR-EPSCs at mf-CA3 synapses without impacting on basal synaptic properties. This decrease in KAR-EPSCs was not accompanied with a detectable increase in extrasynaptic KARs, suggesting that unstabilized synaptic KARs may undergo rapid endocytosis. Although N-cadherin binds AMPARs through an interaction with the N-terminal extracellular domain of GluA2 (ref. 56), or intracellularly^57^, AMPARs were surprisingly not affected by the removal of N-cadherin in our experimental conditions. This was indicated by the lack of effect of N-cadherin inactivation on the amplitude of evoked AMPAR-EPSCs, mEPSCs and AMPAR currents induced by focal glutamate uncaging. Possibly, AMPARs may be stabilized at the PSD in mature synapses via additional transsynaptic adhesion molecules, such as neuroligins or leucine-rich repeat transmembrane (LRRTM) proteins. Hence, our data indicate that N-cadherins specifically recruit and/or stabilize KARs at mf-CA3 synapses.

Synaptic specificity is probably mediated by a network of synaptic molecules. N-cadherins appear to be one of the partner molecules responsible for the synaptic stabilization of KARs at mf-CA3 synapses. Accordingly, it is interesting to note that N-cadherin labelling is particularly intense in the dendritic shaft of CA3 PCs in the stratum lucidum^58^. Other partners are likely to play a role in the subcellular segregation of KARs at these particular synaptic sites. Synaptic specificity in the brain involves differential expression of type II cadherin genes^55^, such as Cadherin-9, which is expressed in both the DG and CA3 PCs, and appears essential for the specific formation of mf-CA3 synapses during development^42^. Cadherin-9 may also play a role in specifying the functional properties of mature mf-CA3 synapses, although this has yet to be explored. An association between N-cadherin and Cadherin-9, possibly assembled as heterodimers, could be essential for the maintenance of specific synaptic features. One can speculate that the loss of Cadherin-9 could similarly impact KAR-EPSCs. Interestingly, a recent report identifies a role for the C1q-like proteins C1ql2 and C1ql3 in the recruitment of KARs at mf-CA3 synapses by direct interaction with the extracellular domain of GluK2 and/or GluK4 subunits. One can speculate that the C1ql2/3 proteins secreted by mfs may regulate the recruitment of KARs and provide input specificity, whereas N-cadherins would stabilize synaptic KARs through indirect interactions with the cytoplasmic domain of GluK2a.

Understanding the molecular mechanisms underlying the synaptic recruitment of KARs at mf-CA3 synapses could also shed light on the pathological process through which aberrant KARs are recruited at recurrent mf onto DG cells in temporal lobe epilepsy^59^. Neutralization of these aberrant KARs markedly attenuates epileptic seizures in a mouse model of temporal lobe epilepsy^60^. The molecular mechanisms underlying synaptic segregation of KARs probably bear similarities at both mf-CA3 synapses and recurrent mf synapses onto DG cells^61^.

In conclusion, multiple mechanisms combine to control the specific subcellular localization of KARs at mf-CA3 synapses, including a stringent control of the amount of GluK2 subunit in CA3 PCs, a limited number of postsynaptic slots for KARs and the recruitment/stabilization of KARs by transsynaptic mechanisms which may involve a coordination between secreted C1ql2/3 proteins, cadherin-9 and N-cadherin.

## Methods

### DNA constructs

GluK2a WT and GluK2a C-terminal mutant cDNAs used in the present study were described previously and were expressed under the control of a cytomegalovirus (CMV) promoter^15^,^38^. DNA plasmids were dissolved at various concentrations in the electroporation solution: GluK2 WT, GluK2 C-terminal mutant and SEP–GluK2 (ref. 62) cDNAs at concentration between 3 and 80 ng μl^−1^; GFP at 30 ng μl^−1^ (CMV); tdTomato, 20 ng μl^−1^ (CMV); NCad-ΔE^63^ at 30 ng μl^−1^ (CMV); GFP-cre at 30 ng μl^−1^ (CMV) and nuclear GFP 30 ng μl^−1^ (CMV). In all uncaging experiments, CA3 PCs were co transfected with GFP or tdTomato to visualize cell morphology. In uncaging experiments, NT refers to cells not transfected with GluK2a (or NCad-ΔE, GFP-cre or nuclear GFP) plasmids, but expressing only GFP or tdTomato. In experiments presented in Figs 4 and 5 and Supplementary Fig. 5e–g, NT refers to CA3 PCs not transfected with any constructs.

### Slice culture preparation and transfection

Organotypic hippocampal slice (300 μm thickness) cultures were prepared from P5 to P7 GluK2^−/−^ and WT FVB mice as described previously^26^, and according to the guidelines of the University of Bordeaux/CNRS Animal Care and Use Committee. Three to 4 days after plating, the medium was replaced and then changed every 2–3 days. After 7 days *in vitro*, visually identified CA3 PCs were transfected by single-cell electroporation. For this, slices were placed in the microscope chamber in the presence of a small volume of a sterile pre-warmed (37 °C) HEPES-based artificial cerebrospinal fluid (ACSF) composed of 145 mM NaCl, 2.5 mM KCl, 2 mM CaCl_2_, 1 mM MgCl_2_, 10 mM HEPES and 10 mM glucose, adjusted to 310 mOsm l^−1^ and pH 7.4 with NaOH at room temperature. Electroporation pipettes (8–10 mΩ) were filled with a solution containing the following: 140 mM CH_3_KSO_3_, 2 mM MgCl_2_, 4 mM NaCl, 5 mM P-creatine, 3 mM Na_2_ATP, 0.33 mM GTP, 0.2 mM EGTA and 10 mM HEPES adjust to 300 mOsm l^−1^ and pH 7.2 with KOH. cDNA was add to the internal solution the day of the experiment. The electroporation pipette was approached to the cell body in loose cell-attached configuration. A train of stimulation to the cell body (50 square pulses, 600 μs duration, at 100 Hz with an amplitude of −11.5 V). The pulses were delivered by a stimulating unit controlled by an EPC9 amplifier (HEKA Elektronik, Lambrecht/Pfalz, Germany) and the pipette was slowly retracted and positioned on an other cell body.

### *In vivo* virus injection and acute slices preparation

Stereotaxic viral injections were performed on 3-week-old male C57bl6 N-Cadherin^fl/fl^ mice. The mice were injected with 300 nl (55 nl min^−1^) of an AAV2/9 encoding for GFP-Cre under a synapsin promotor (AAV2/9.hsynapsin.hghintron.GFP.cre.wpre.sv40; titer 1.33^11^) in the CA3 area of the right hippocampus with the following coordinates: +1.90 mm mediolateral (ML), −1.90 mm anteroposterior (AP) and −1.9 mm dorsoventral (DV). For acute slices recordings, transverse hippocampal slices (320 μm thick) were obtained from 17- to 19-day-old FVB GluK2^−/−^ and GluK2 ^+/+^ mice and from 2-month-old N-Cadherin^fl/fl^ mice anaesthetized with ketamine (75 mg kg^−1^) and xylazine (10 mg kg^−1^) mix, and killed by decapitation.

### Electrophysiology

Whole-cell patch-clamp experiments were performed at 33 °C from CA3 PCs expressing or not GFP 3–4 days after transfection. Whole-cell voltage-clamp recordings were performed with 3–4 MΩ electrodes filled with a solution containing 140 mM CsCH_3_SO_3_, 2 mM MgCl_2_, 4 mM NaCl, 5 mM P-creatine, 3 mM Na_2_ATP, 0.33 mM GTP, 0.2 mM EGTA and 10 mM HEPES adjust to 300 mOsm l^−1^ and pH 7.2 with CsOH. In experiments in which mf or A/C inputs were electrically stimulated by an extracellular electrode, slices were perfused with an extracellular solution (ACSF) composed of: 125 mM NaCl, 2.5 mM KCl, 1.25 mM NaH_2_PO_4_, 26 mM NaHCO_3_, 2.3 mM CaCl_2_, 1.3 mM MgCl_2_ and 25 mM glucose saturated with 95% O_2_/5% CO_2_. Mf synaptic responses were evoked by minimal stimulation^64^ by placing a patch pipette filled with aCSF in the hilus of the DG. A/C excitatory stimulations were evoked by placing the pipette in the stratum radiatum above the CA3 PC layer. The distinct nature of the stimulated inputs was confirmed by recording short-term plasticity properties. These experiments were performed at 30–32 °C in the presence of pharmacological drugs in the aCSF: NBQX 150 nM, DAP-5 50 μM and bicuculline 10 μM. For experiments with synaptic stimulation, one cell was recorded per slice. KAR currents were pharmacologically isolated in the presence of LY 303070 25 μM, a selective AMPAR antagonist. For glutamate uncaging experiments, slices were bathed with 2 ml of HEPES-based ACSF (room temperature) composed of 140 mM NaCl, 3.5 mM KCl, 6 mM HEPES, 4 mM Na-HEPES, 2.3 mM CaCl_2_, 1.3 mM MgCl_2,_ 10 mM glucose and 1 mM sodium pyruvate. One to three cells were recorded per slice. Glutamate receptors were activated by glutamate uncaging in the presence of MNI glutamate 500 μM (2 ms duration at 0.1 Hz). Glutamate uncaging was performed on a Nikon FN1 microscope equipped with a × 60 lens (numerical aperture 1.00) and a 405 nm Obis laser (Coherent, Germany; 50 mW CW) controlled by a Digitimer DS2A (Digitimer, Welwiyn, UK).

The access resistance was <20 MΩ and cells were discarded if it changed by >20%. No series resistance compensation was used. Recordings were made using EPC 9 or EPC10 amplifiers (HEKA Elektronik, Lambrecht/Pfalz, Germany), were filtered at 0.5–1 kHz, digitalized at 5 kHz and stored on a personal computer for further analysis (IGOR PRO 6.2; WaveMetrics, Lake Oswego, OR). Values are presented as mean ±s.e.m. Electrophysiological analysis was performed with the IGOR PRO software through NeuroMatic v2.6 plugin. The decaying phase of the currents was fitted with a function of the following form: *y*(*t*)=Σ*A*_i_ exp(−*t*/*τ*_i_), where *A*_i_ is the fraction of respective components (Σ*A*_i_=1) and *τ*_i_ is the time constant *τ*_ω_ calculated using the formula *τ*_ω_=Σ*A*_i_ *τ*_i_. A maximum of two exponential components was used. It has been shown that in organotypic slice cultures A/C fast synaptic transmission, unlike mf-CA3, can be in part mediated by P2X receptors activated by ATP^3^,^65^. We have observed similar residual currents in our preparation both in the presence of NBQX 25 μM and in GluK2^−/−^ animals (data not shown). For this reason, to quantify KAR-mediated EPSCs at A/C input we have subtracted the residual current after application of LY 30,3070, with the residual current after the further application of NBQX (to block AMPARs and KARs).

### Immunohistochemistry and SEP–GluK2a

Live organotypic hippocampal slices in which CA3-PCs were electroporated with TdTomato and SEP–GluK2a were labelled with 200 nM GFP-nanobody coupled to ATTO647N (ATTO-TEC)^36^ in a dark chamber for 30–60 min at room temperature in the same ACSF used for uncaging experiments supplemented with 1% globulin-free BSA (Sigma. St Louis, MO). Slices were then rinsed and fixed in 4% paraformaldehyde (PFA) containing 20% sucrose for 2 h at 4 °C, before rinsing with PBS and mounting in mowiol 4–88 (Sigma). Confocal images were acquired sequentially on a commercial Leica TCS SPE microscope equipped with three laser diodes (488, 561, 647 nm) using a × 63 oil objective and a pinhole opened to one time the Airy disk. The number of SEP–GluK2 clusters per micrometre was determined using Metamorph software (Molecular Devices, USA). Briefly, *z*-projections of the stacks in the 488 (SEP) and 635 (ATTO647N) channels were segmented after background subtraction. Segmented images were overlaid and GluK2 clusters were defined as individual spots that were present in both channels and co-localized within determined areas of the neuron (soma, TEs and DDs).

In Fig. 1 and Supplementary Fig. 1, the confocal images were obtained after fixation of organotypic slices following glutamate uncaging on organotypic slices from WT FVB mice electroporated with a cDNA construct coding for GFP-N1. The slices were fixed with PFA, 30% sucrose for 15 min at room temperature. After blocking within PBS, 10% fetal calf serum for 1 h, we immunodetected GFP by incubating the slices with a mouse monoclonal antibody anti-GFP (Roche; dilution 1/200, overnight at 4 °C) in PBS containing 0.2% Triton and 5% SVF. After washing three times in PBS, the slices were incubated with a fluorophore-coupled secondary antibody anti-mouse (Life Technologies) in PBS containing 5% SVF and 0.2% Triton (dilution 1:1,000, for 2 h at room temperature). Finally, the slices were washed three times at room temperature with PBS and coverslipped in Vectashield medium (Vector).

### Confocal laser-scanning fluorescence microscopy and image analysis

Confocal image stacks of PFA-fixed organotypic slices were acquired with a Leica DM6000 TCQS SP8 X white laser-scanning microscope using the objective Leica HCX PL Apo CS × 100 oil numerical aperture 1.4. Images were processed with ImageJ 1.49d software (Wayne Rasband, National Institutes of Health, USA). Illustrations correspond to two-dimensional maximal projections of three-dimensional acquired confocal stacks smoothened by the median filter.

### Drugs

All drugs were obtained from Tocris Cookson (Bristol, UK), Sigma or Ascent Scientific (Bristol, UK).

### Data availability

The data that support the findings of this study are available from the corresponding author on request.

## Additional information

**How to cite this article:** Fièvre, S. *et al.* Molecular determinants for the strictly compartmentalized expression of kainate receptors in CA3 pyramidal cells. *Nat. Commun.* 7:12738 doi: 10.1038/ncomms12738 (2016).

## Supplementary Material

Supplementary Information Supplementary Figures 1-5.

## References

[b1] Williams M. E., de Wit J. & Ghosh A. Molecular mechanisms of synaptic specificity in developing neural circuits. Neuron 68, 9–18 (2010). 20920787 10.1016/j.neuron.2010.09.007 PMC3327884

[b2] Henze D. A., Urban N. N. & Barrionuevo G. The multifarious hippocampal mossy fiber pathway: a review. Neuroscience 98, 407–427 (2000). 10869836 10.1016/s0306-4522(00)00146-9

[b3] Nicoll R. A. & Schmitz D. Synaptic plasticity at hippocampal mossy fibre synapses. Nat. Rev. Neurosci. 6, 863–876 (2005). 16261180 10.1038/nrn1786

[b4] Paoletti P., Bellone C. & Zhou Q. NMDA receptor subunit diversity: impact on receptor properties, synaptic plasticity and disease. Nat. Rev. Neurosci. 14, 383–400 (2013). 23686171 10.1038/nrn3504

[b5] Bettler B. & Mulle C. Review: neurotransmitter receptors. II. AMPA and kainate receptors. Neuropharmacology 34, 123–139 (1995). 7542368 10.1016/0028-3908(94)00141-e

[b6] Contractor A., Mulle C. & Swanson G. T. Kainate receptors coming of age: milestones of two decades of research. Trends Neurosci. 34, 154–163 (2011). 21256604 10.1016/j.tins.2010.12.002 PMC3051042

[b7] Lerma J. & Marques J. M. Kainate receptors in health and disease. Neuron 80, 292–311 (2013). 24139035 10.1016/j.neuron.2013.09.045

[b8] Rodríguez-Moreno A. & Sihra T. S. Kainate receptors with a metabotropic modus operandi. Trends Neurosci. 30, 630–637 (2007). 17981346 10.1016/j.tins.2007.10.001

[b9] Carta M., Fièvre S., Gorlewicz A. & Mulle C. Kainate receptors in the hippocampus. Eur. J. Neurosci. 39, 1835–1844 (2014). 24738709 10.1111/ejn.12590

[b10] Castillo P. E., Malenka R. C. & Nicoll R. A. Kainate receptors mediate a slow postsynaptic current in hippocampal CA3 neurons. Nature 388, 182–186 (1997). 9217159 10.1038/40645

[b11] Mulle C. *et al.* Altered synaptic physiology and reduced susceptibility to kainate-induced seizures in GluR6-deficient mice. Nature 392, 601–605 (1998). 9580260 10.1038/33408

[b12] Ruiz A., Sachidhanandam S., Utvik J. K., Coussen F. & Mulle C. Distinct subunits in heteromeric kainate receptors mediate ionotropic and metabotropic function at hippocampal mossy fiber synapses. J. Neurosci. 25, 11710–11718 (2005). 16354929 10.1523/JNEUROSCI.4041-05.2005 PMC6726035

[b13] Darstein M., Petralia R. S., Swanson G. T., Wenthold R. J. & Heinemann S. F. Distribution of kainate receptor subunits at hippocampal mossy fiber synapses. J. Neurosci. 23, 8013–8019 (2003). 12954862 10.1523/JNEUROSCI.23-22-08013.2003 PMC6740495

[b14] Garcia E. P. *et al.* SAP90 binds and clusters kainate receptors causing incomplete desensitization. Neuron 21, 727–739 (1998). 9808460 10.1016/s0896-6273(00)80590-5

[b15] Coussen F. *et al.* Co-assembly of two GluR6 kainate receptor splice variants within a functional protein complex. Neuron 47, 555–566 (2005). 16102538 10.1016/j.neuron.2005.06.033

[b16] Coussen F. *et al.* Recruitment of the kainate receptor subunit glutamate receptor 6 by cadherin/catenin complexes. J. Neurosci. 22, 6426–6436 (2002). 12151522 10.1523/JNEUROSCI.22-15-06426.2002 PMC6758172

[b17] Copits B. A. & Swanson G. T. Kainate receptor post-translational modifications differentially regulate association with 4.1N to control activity-dependent receptor endocytosis. J. Biol. Chem. 288, 8952–8965 (2013). 23400781 10.1074/jbc.M112.440719 PMC3610968

[b18] Zhang W. *et al.* A transmembrane accessory subunit that modulates kainate-type glutamate receptors. Neuron 61, 385–396 (2009). 19217376 10.1016/j.neuron.2008.12.014 PMC2803770

[b19] Copits B. A., Robbins J. S., Frausto S. & Swanson G. T. Synaptic targeting and functional modulation of GluK1 kainate receptors by the auxiliary neuropilin and Tolloid-like (NETO) proteins. J. Neurosci. 31, 7334–7340 (2011). 21593317 10.1523/JNEUROSCI.0100-11.2011 PMC3131203

[b20] Straub C. *et al.* Distinct functions of kainate receptors in the brain are determined by the auxiliary subunit Neto1. Nat. Neurosci. 14, 866–873 (2011). 21623363 10.1038/nn.2837 PMC3125417

[b21] Fisher J. L. & Mott D. D. The auxiliary subunits Neto1 and Neto2 reduce voltage-dependent inhibition of recombinant kainate receptors. J. Neurosci. 32, 12928–12933 (2012). 22973017 10.1523/JNEUROSCI.2211-12.2012 PMC3652014

[b22] Tang M. *et al.* Neto1 is an auxiliary subunit of native synaptic kainate receptors. J. Neurosci. 31, 10009–10018 (2011). 21734292 10.1523/JNEUROSCI.6617-10.2011 PMC3148853

[b23] Copits B. A. & Swanson G. T. Dancing partners at the synapse: auxiliary subunits that shape kainate receptor function. Nat. Rev. Neurosci. 13, 675–686 (2012). 22948074 10.1038/nrn3335 PMC3520510

[b24] Palacios-Filardo J., Aller M. I. & Lerma J. Synaptic targeting of kainate receptors. Cerebral Cortex 26, 1464–1472 (2014). 25316333 10.1093/cercor/bhu244

[b25] Benson D. L. & Huntley G. W. Synapse adhesion: a dynamic equilibrium conferring stability and flexibility. Curr. Opin. Neurobiol. 22, 397–404 (2012). 22019151 10.1016/j.conb.2011.09.011 PMC3492943

[b26] Carta M. *et al.* CaMKII-dependent phosphorylation of GluK5 mediates plasticity of kainate receptors. EMBO J. 32, 496–510 (2013). 23288040 10.1038/emboj.2012.334 PMC3579137

[b27] Trigo F. F., Corrie J. E. T. & Ogden D. Laser photolysis of caged compounds at 405nm: Photochemical advantages, localisation, phototoxicity and methods for calibration. J. Neurosci. Methods 180, 9–21 (2009). 19427524 10.1016/j.jneumeth.2009.01.032

[b28] Rollenhagen A. *et al.* Structural determinants of transmission at large hippocampal mossy fiber synapses. J. Neurosci. 27, 10434–10444 (2007). 17898215 10.1523/JNEUROSCI.1946-07.2007 PMC6673150

[b29] Wilke S. A. *et al.* Specific disruption of hippocampal mossy fiber synapses in a mouse model of familial Alzheimer's disease. PLoS ONE 9, e84349 (2014). 24454724 10.1371/journal.pone.0084349 PMC3890281

[b30] Contractor A. *et al.* Loss of kainate receptor-mediated heterosynaptic facilitation of mossy-fiber synapses in KA2−/− mice. J. Neurosci. 23, 422–429 (2003). 12533602 10.1523/JNEUROSCI.23-02-00422.2003 PMC6741894

[b31] Barberis A., Sachidhanandam S. & Mulle C. GluR6/KA2 kainate receptors mediate slow-deactivating currents. J. Neurosci. 28, 6402–6406 (2008). 18562611 10.1523/JNEUROSCI.1204-08.2008 PMC6670893

[b32] Fernandes H. B. *et al.* High-affinity kainate receptor subunits are necessary for ionotropicbut not metabotropic signaling. Neuron 63, 818–829 (2009). 19778510 10.1016/j.neuron.2009.08.010 PMC2756730

[b33] Pinheiro P. S. *et al.* Selective block of postsynaptic kainate receptors reveals their function at hippocampal mossy fiber synapses. Cereb. Cortex 23, 323–331 (2013). 22345355 10.1093/cercor/bhs022

[b34] González-González I. M. & Henley J. M. Postsynaptic kainate receptor recycling and surface expression are regulated by metabotropic autoreceptor signalling. Traffic 14, 810–822 (2013). 23556457 10.1111/tra.12071 PMC3744763

[b35] Ries J., Kaplan C., Platonova E., Eghlidi H. & Ewers H. A simple, versatile method for GFP-based super-resolution microscopy via nanobodies. Nat. Methods 9, 582–584 (2012). 22543348 10.1038/nmeth.1991

[b36] Chamma I. *et al.* Mapping the dynamics and nanoscale organization of synaptic adhesion proteins using monomeric streptavidin. Nat. Commun. 7, 10773 (2016). 26979420 10.1038/ncomms10773 PMC4799371

[b37] Hirbec H. *et al.* Rapid and differential regulation of AMPA and kainate receptors at hippocampal mossy fibre synapses by PICK1 and GRIP. Neuron 37, 625–638 (2003). 12597860 10.1016/s0896-6273(02)01191-1 PMC3314502

[b38] Jaskolski F. *et al.* Subunit composition and alternative splicing regulate membrane delivery of kainate receptors. J. Neurosci. 24, 2506–2515 (2004). 15014126 10.1523/JNEUROSCI.5116-03.2004 PMC6729486

[b39] Yan S. *et al.* A C-terminal determinant of GluR6 kainate receptor trafficking. J. Neurosci. 24, 679–691 (2004). 14736854 10.1523/JNEUROSCI.4985-03.2004 PMC6729259

[b40] Chazeau A. *et al.* Mechanical coupling between transsynaptic N-cadherin adhesions and actin flow stabilizes dendritic spines. Mol. Biol. Cell 26, 859–873 (2015). 25568337 10.1091/mbc.E14-06-1086 PMC4342023

[b41] Riehl R. *et al.* Cadherin function is required for axon outgrowth in retinal ganglion cells *in vivo*. Neuron 17, 837–848 (1996). 8938117 10.1016/s0896-6273(00)80216-0

[b42] Williams M. E. *et al.* Cadherin-9 regulates synapse-specific differentiation in the developing hippocampus. Neuron 71, 640–655 (2011). 21867881 10.1016/j.neuron.2011.06.019 PMC3272880

[b43] Li J. *et al.* Cardiac-specific loss of N-cadherin leads to alteration in connexins with conduction slowing and arrhythmogenesis. Circ. Res. 97, 474–481 (2005). 16100040 10.1161/01.RES.0000181132.11393.18

[b44] Beed P. S., Salmen B. & Schmitz D. GluK2-mediated excitability within the superficial layers of the entorhinal cortex. PLoS ONE 4, e5576 (2009). 19440371 10.1371/journal.pone.0005576 PMC2679203

[b45] Huang Y. H., Dykes-Hoberg M., Tanaka K., Rothstein J. D. & Bergles D. E. Climbing fiber activation of EAAT4 transporters and kainate receptors in cerebellar Purkinje cells. J. Neurosci. 24, 103–111 (2004). 14715943 10.1523/JNEUROSCI.4473-03.2004 PMC6729555

[b46] Wilke S. A. *et al.* Deconstructing complexity: serial block-face electron microscopic analysis of the hippocampal mossy fiber synapse. J. Neurosci. 33, 507–522 (2013). 23303931 10.1523/JNEUROSCI.1600-12.2013 PMC3756657

[b47] Bureau I., Dieudonne S., Coussen F. & Mulle C. Kainate receptor-mediated synaptic currents in cerebellar Golgi cells are not shaped by diffusion of glutamate. Proc. Natl Acad. Sci. USA 97, 6838–6843 (2000). 10841579 10.1073/pnas.97.12.6838 PMC18759

[b48] Choquet D. & Triller A. Perspective. Neuron 80, 691–703 (2013). 24183020 10.1016/j.neuron.2013.10.013

[b49] Granger A. J., Shi Y., Lu W., Cerpas M. & Nicoll R. A. LTP requires a reserve pool of glutamatereceptors independent of subunit type. Nature 493, 495–500 (2013). 23235828 10.1038/nature11775 PMC3998843

[b50] Martin S., Nishimune A., Mellor J. R. & Henley J. M. SUMOylation regulates kainate-receptor-mediated synaptic transmission. Nature 447, 321–325 (2007). 17486098 10.1038/nature05736 PMC3310901

[b51] Maraschi A. *et al.* Parkin regulates kainate receptors by interacting with the GluK2 subunit. Nat. Commun. 5, 5182 (2014). 25316086 10.1038/ncomms6182 PMC4218952

[b52] Selak S. *et al.* A role for SNAP25 in internalization of kainate receptors and synaptic plasticity. Neuron 63, 357–371 (2009). 19679075 10.1016/j.neuron.2009.07.017

[b53] Chamberlain S. E. L. *et al.* SUMOylation and phosphorylation of GluK2 regulate kainate receptor trafficking and synaptic plasticity. Nat. Neurosci. 15, 845–852 (2012). 22522402 10.1038/nn.3089 PMC3435142

[b54] Suzuki E. & Kamiya H. PSD-95 regulates synaptic kainate receptors at mouse hippocampal mossy fiber-CA3 synapses. Neurosci. Res. 107, 14–19 (2016). 26746114 10.1016/j.neures.2015.12.011

[b55] Basu R., Taylor M. R. & Williams M. E. The classic cadherins in synaptic specificity. Cell. Adh. Migr. 9, 193–201 (2015). 25837840 10.1080/19336918.2014.1000072 PMC4594527

[b56] Saglietti L. *et al.* Extracellular interactions between GluR2 and N-cadherin in spine regulation. Neuron 54, 461–477 (2007). 17481398 10.1016/j.neuron.2007.04.012

[b57] Nuriya M. & Huganir R. L. Regulation of AMPA receptor trafficking by N-cadherin. J. Neurochem. 97, 652–661 (2006). 16515543 10.1111/j.1471-4159.2006.03740.x

[b58] Fannon A. M. & Colman D. R. A model for central synaptic junctional complex formation based on the differential adhesive specificities of the cadherins. Neuron 17, 423–434 (1996). 8816706 10.1016/s0896-6273(00)80175-0

[b59] Epsztein J., Represa A., Jorquera I., Ben-Ari Y. & Crépel V. Recurrent mossy fibers establish aberrant kainate receptor-operated synapses on granule cells from epileptic rats. J. Neurosci. 25, 8229–8239 (2005). 16148230 10.1523/JNEUROSCI.1469-05.2005 PMC6725550

[b60] Peret A. *et al.* Contribution of aberrant GluK2-containing kainate receptors to chronic seizuresin temporal lobe epilepsy. Cell Rep. 8, 347–354 (2014). 25043179 10.1016/j.celrep.2014.06.032

[b61] Matsuda K. *et al.* Transsynaptic modulation of kainate receptor functions by C1q-like proteins. Neuron 90, 752–767 (2016). 27133466 10.1016/j.neuron.2016.04.001

[b62] Martin S., Bouschet T., Jenkins E. L., Nishimune A. & Henley J. M. Bidirectional regulation of kainate receptor surface expression in hippocampal neurons. J. Biol. Chem. 283, 36435–36440 (2008). 18955488 10.1074/jbc.M806447200 PMC2662304

[b63] Bard L. *et al.* A molecular clutch between the actin flow and N-cadherin adhesions drives growth cone migration. J. Neurosci. 28, 5879–5890 (2008). 18524892 10.1523/JNEUROSCI.5331-07.2008 PMC6670336

[b64] Marchal C. & Mulle C. Postnatal maturation of mossy fibre excitatory transmission in mouse CA3 pyramidal cells: a potential role for kainate receptors. J. Physiol. (Lond.) 561, 27–37 (2004). 15358807 10.1113/jphysiol.2004.069922 PMC1665334

[b65] Mori M., Heuss C., Gähwiler B. H. & Gerber U. Fast synaptic transmission mediated by P2X receptors in CA3 pyramidal cells of rat hippocampal slice cultures. J. Physiol. (Lond.) 535, 115–123 (2001). 11507162 10.1111/j.1469-7793.2001.t01-1-00115.x PMC2278762

