# Supplementary material for: Molecular determinants for the strictly compartmentalized expression of kainate receptors in CA3 pyramidal cells
Source: Nat Commun. 2016 Sep 27;7:12738. doi: 10.1038/ncomms12738 (PMC5052629; doi:10.1038/ncomms12738)
Supplement: Supplementary Information — Supplementary Figures 1-5. [file ncomms12738-s1.pdf]

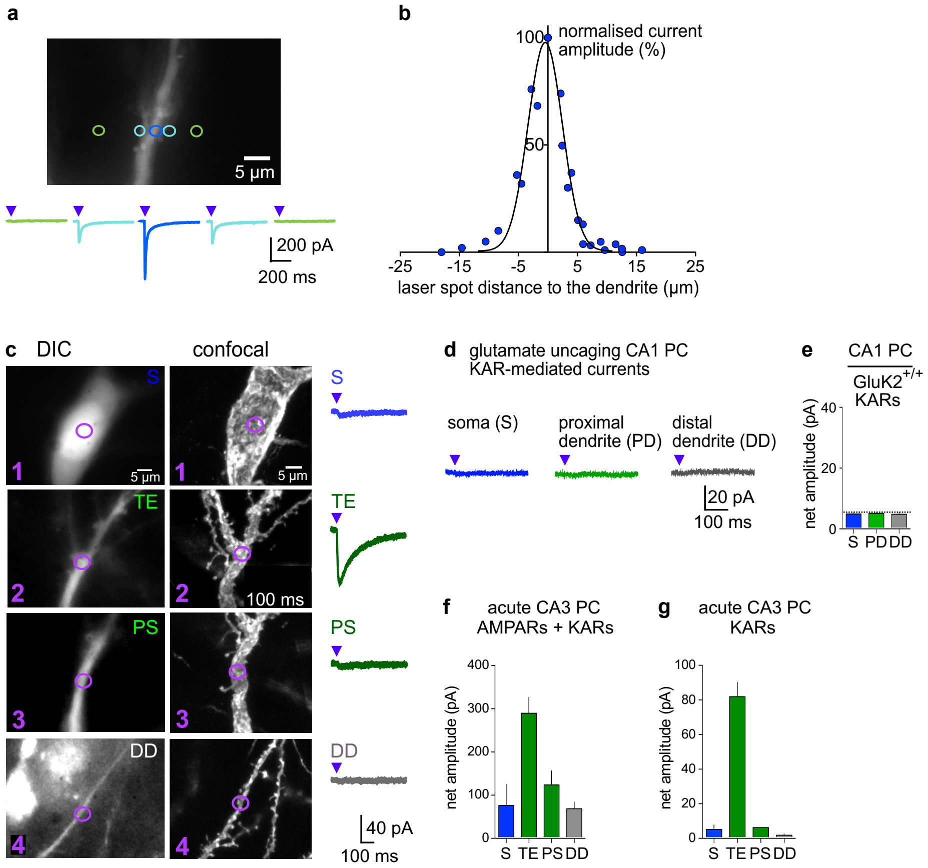

Supplementary Figure 1: Spatial precision of monophoton glutamate uncaging in organotypic slice cultures. **a**) To have access to the spatial precision of glutamate uncaging system, AMPARs-currents were recorded following glutamate uncaging at different distances from a dendrite. **b**) Graph representation of the percentage of current amplitude after uncaging on the dendrite to the current amplitude after uncaging at different distance from the dendrite itself. **c**) Epifluorescence images and corresponding confocal images of a CA3 PC (GluK2$^{+/+}$) illustrating the location where glutamate uncaging (MNI-glutamate 500 µM) was performed and the corresponding evoked KAR-current (the image of the cell was already used in Figure 1). Purple arrow heads represent the time at which uncaging was done (405 nm, 2 ms). Glutamate uncaging was performed on the soma (S), thorny excrescences (TE), proximal shaft (PS) and distal dendrite (DD). The confocal images were acquired after glutamate uncaging, fixation and mounting of the slice. **d, e**) Sample traces illustrating KAR-currents evoked by glutamate uncaging (in the presence of 25 µM LY303070) in organotypic slices prepared from GluK2$^{+/+}$ mice. Uncaging was performed in different districts of the CA1 PC (soma, proximal dendrite and distal dendrite). Summary bar graph illustrating the average net amplitude of the evoked currents (net amplitude soma: 4.5 ± 0.3 pA; proximal dendrite: 4.7 ± 0.2 pA; distal dendrite: 4.4 ± 0.6 pA; n=5)**. f, g)** Bar graph summarizing the average net amplitude of evoked currents after glutamate uncaging in different compartments of CA3 PCs (S, TE, PD, DD) from acute (GluK2$^{+/+}$) slices in absence of LY303070 (F) in order to record both the AMPAR and KAR component (net amplitude: S: 73.5 pA ± 49.1 pA; TE: 286.7 ± 37.1 pA; PS: 121.2 ± 32.6 pA; DD: 65.7 ± 15.6 pA; n=4) or in presence of LY 303070 (G) in order to record KARs component (net amplitude: S: 4.4 ± 2.7 pA; TE: 81.23 ± 8.3 pA; PS: 5.5 ± 0.0 pA; 1.0 ± 0.7 pA n=4). In all panels, values are presented as mean ± SEM of n experiments.

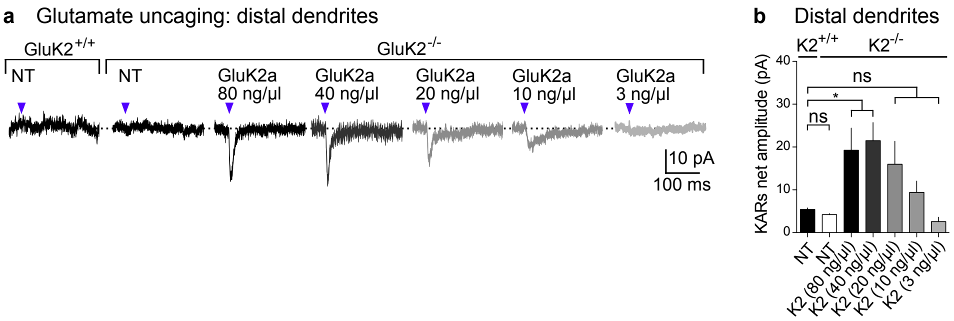

Supplementary Figure 2: Semi-quantitative replacement of KARs. **a)** Sample traces illustrating the KAR-currents evoked by glutamate uncaging on the distal dendrites CA3 PCs in organotypic slice cultures prepared from GluK2+/+ and GluK2-/- not transfected (NT) or transfected with different amount of GluK2a cDNA (80, 40, 20, 10, 3 ng/µl). **b)** Summary bar graphs illustrating the average amplitude of the KARs evoked currents at the distal dendrites (Average net current amplitude: WT: 5.4 ± 0.3 pA; GluK2a 80 ng/µl: 19.2 ± 5.2 pA; GluK2a 40 ng/µl: 21.5 ± 4.2 pA; GluK2a 20 ng/µl: 16.0 ± 5.4 pA; GluK2a 10 ng/µl: 9.4 ± 2.6 pA; GluK2a 3 ng/µl: 2.6 ± 1.0 pA). In all panels, values are presented as mean ± SEM of n experiments. Data were compared using one way ANOVA followed by Dunnett's multiple comparison test (ns $p>0.05$, *$p<0.05$).

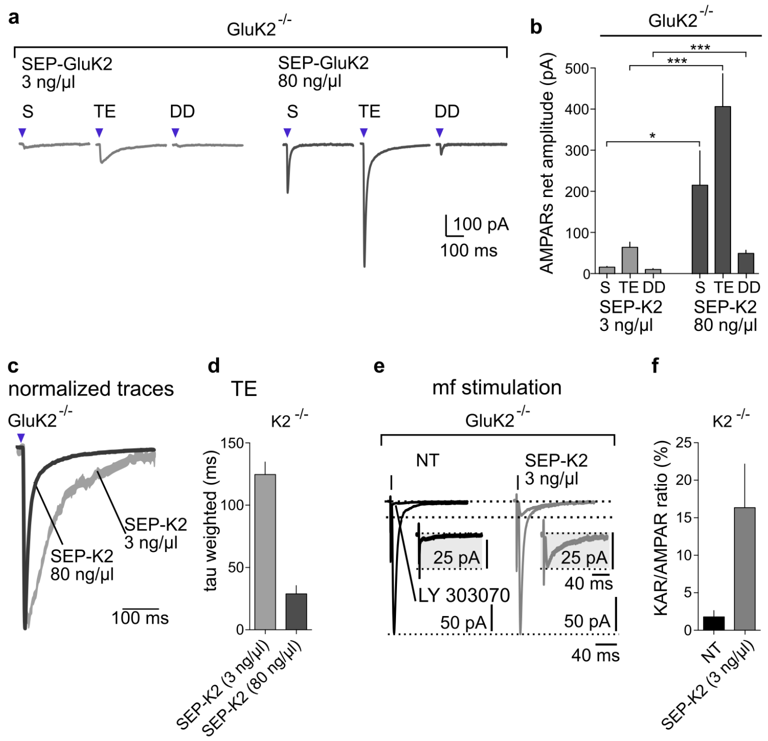

Supplementary Figure 3: Rescue of KAR-currents with GluK2a-SEP. **a)** Sample traces illustrating KAR-currents evoked by glutamate uncaging in different districts of CA3 PCs (S, TE and DD) in organotypic slice cultures prepared from GluK2-/- mice and transfected with either 3ng/µl or 80 ng/µl of GluK2a-SEP cDNA. **b)** Summary bar graphs illustrating the average amplitude of KAR-currents illustrated in (a). GluK2a-Sep 3 ng/µl; S, 17.1 ± 2.3 pA; TE, 63.9 ± 12.7 pA; DD, 9.7 ± 2.6 pA, experiments performed on 6-7 cells, 4 slices and 3 mice. GluK2a-Sep 80 ng/µl; mean amplitude, soma, 216.5 ± 83.6 pA; TE, 406.3 ± 80.2 pA; DD, 49.4 ± 7.5 pA; experiments performed on 5-6 cells, 4 slices and 3 mice. **c, d)** Sample traces of KAR-currents evoked by glutamate uncaging showed in a, and scaled at the peak illustrating differences in the decay between SEP-GluK2a transfected at 3 ng/µl or 80 ng/µl. Quantification of the decay rate (tau weighted) of KAR-currents evoked onto TE by glutamate uncaging for SEP-GluK2a transfected at 3 ng/µl (mean tau weighted, 28.8 ± 6.7 ms, n= 7) or 80 ng/µl (mean tau weighted, 124.7 ± 9.9 ms, n =6). **e, f)** Sample traces illustrating synaptic currents evoked by mf stimulation in organotypic slices prepared from GluK2$^{-/-}$ mice, not transfected (NT) or transfected with SEP-GluK2a cDNA 3 ng/µl. Experiments were performed in the presence of bicuculline (10 µ M), D-AP5 (50 µM) and NBQX (150 nM). Traces represent the average synaptic currents evoked at 3 Hz in the absence and in the presence of 25 µM LY303070, to isolate the AMPAR and the KAR component, respectively. In the insert are showed enlargements, by the same proportion, of isolated KAR-EPSCs. Bar graph summarizing the KAR/AMPAR ratio obtained from the experiments presented in e. GluK2$^{-/-}$ NT: 1.7 ± 0.8 %, experiment performed on 3 cells, 3 slices and 2 mice; 3 ng/µl: 16.35 ± 5.8 %, experiment performed on 6 cells, 6 slices and 3 mice. In all panels, values are presented as mean ± SEM of n experiments. Data were compared using unpaired t-test (ns p>0.05, *p<0.05, ***p<0.001).

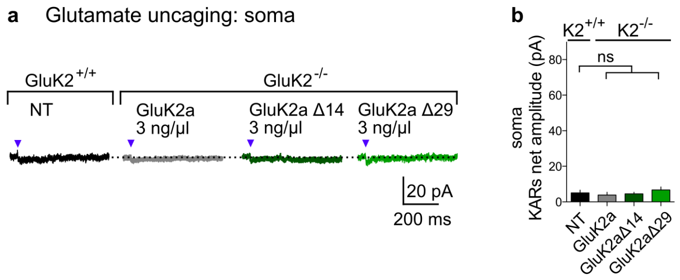

Supplementary Figure 4: Glutamate uncaging on the soma after reexpression of CTD truncated GluK2 subunits. **a)** Sample traces illustrating the KAR-currents evoked by glutamate uncaging on soma of CA3 PCs in organotypic slice cultures prepared from GluK2$^{+/+}$ and GluK2$^{-/-}$ not transfected (NT) or transfected with 3 ng/µl of wt or mutated GluK2a cDNA (Δ14 and Δ29).**b)** Summary bar graphs illustrating the average amplitude of the KARs evoked currents presented in (A) (Average net current amplitude: WT: 5.0 ± 1.6 pA; GluK2a: 3.8 ± 1.6 pA; GluK2a Δ14: 4.5 ± 0.9 pA; GluK2a Δ29: 6.7 ± 1.7 pA; GluK2$^{-/-}$, not transfected 1.0 ± 0.1 pA n=7-10). Values are presented as mean ± SEM of n experiments. Data were compared using one way ANOVA followed by Dunnett's multiple comparison test (ns p>0.05).

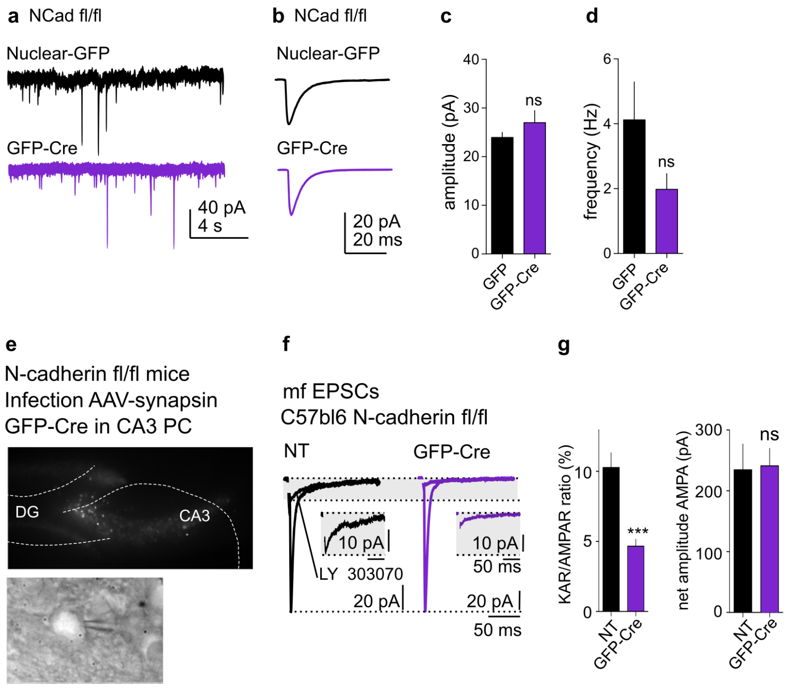

Supplementary Figure 5: Effect of N-cadherin knockout on mEPSCs in organotypic cultures; *in vivo* knockout of N-Cadherin in CA3 PC decreases the amount of KARs at mf-CA3 synapse. **a, b)** Sample traces illustrating mEPSCs recorded from CA3 PCs in organotypic slice cultures prepared from NCad$^{flox/flox}$ mice electroporated either with nuclear-GFP or GFP-Cre. Experiments were performed -70 mV, in the presence of TTX, bicuculline and AP5 at 34 °C. **c, d)** Bar graphs summarizing the average amplitude and frequency of mEPSCs from experiments presented in (a) and (b). Nuclear-GFP, mean amplitude, 23.9 ± 1.1 pA; mean frequency, 4.1 ± 1.1 Hz; Experiment performed on 6 cells, 4 slices and 3 mice. GFP-Cre, mean amplitude, 26.9 ± 2.5 pA; mean frequency, 1.9 ± 0.5 Hz; Experiment performed on 6 cells, 4 slices and 3 mice. **e)** Representative hippocampal acute slice from N-Cadherin$^{fl/fl}$ mice infected in the CA3 region with an AAV2.9 encoding GFP-cre. Slices were prepared 3-4 weeks after infection. **f)** Sample traces illustrating synaptic currents evoked by mf stimulation in CA3 PCs from acute slices prepared from N-Cadherin$^{fl/fl}$ mice not transfected (NT) or infected with GFP-Cre (for clarity stimulus artifacts were removed). Experiments were carried in the presence of bicuculline (10 μM), D-AP5 (50 μM) and NBQX (150 nM). Traces represent the average of synaptic currents evoked at 3 Hz in the absence and in the presence of 25 μM LY303070, to isolate the AMPAR and the KAR component, respectively. In the insert are showed enlargements, by the same proportion, of isolated KAR-EPSCs. **g)** Summary bar graphs illustrating the KAR/AMPAR and the amplitude of the AMPAR EPSCs obtained from the experiments presented in B. (mean KAR/AMPAR ratio: not transfected: 10.3 ± 1.0 %; GFP-cre: 4.7 ± 0.5 %; n=13; mean amplitude: not transfected: 234.6 ± 42.4 pA; GFP-cre: 241.0 ± 28.9 pA; n=13). In all panels, values are presented as mean ± SEM of n experiments. Data were compared using unpaired t-test (ns p>0.05, **p<0.01).
